# No impact of helminth coinfection in patients with smear positive tuberculosis on immunoglobulin levels using a novel method measuring *Mycobacterium tuberculosis*-specific antibodies

**DOI:** 10.1186/s13223-023-00808-0

**Published:** 2023-06-29

**Authors:** Giggil Pushpamithran, Camilla Skoglund, Fanny Olsson, Melissa Méndez-Aranda, Thomas Schön, Mårten Segelmark, Olle Stendahl, Robert H. Gilman, Robert Blomgran

**Affiliations:** 1 Division of Inflammation and Infection, Department of Biomedical and Clinical Sciences, Faculty of Medicine and Health Sciences, Linköping University Campus US, Building 420 Floor 12, 581 85 Linköping, SE Sweden; 2 Division of Clinical Chemistry and Pharmacology, Department of Biomedical and Clinical Sciences, Faculty of Medicine and Health Sciences, Linköping University, Linköping, Sweden; 3 Laboratorio de Investigación en Enfermedades Infecciosas, LID, Universidad Peruana Cayetano Heredia, Lima, Peru; 4 Department of Clinical Sciences, Lund University and Department of Nephrology, Skane University Hospital, Lund, Sweden; 5 Department of International Health, Johns Hopkins School of Public Health, Baltimore, Mayland USA; 6 Department of Infectious Diseases, County of Östergötland and Kalmar, Linköping University, Linköping, Sweden

**Keywords:** *Mycobacterium tuberculosis*, Active pulmonary TB, Helminth coinfection, Adaptive humoral immune response, Mtb-specific IgG IgA IgM

## Abstract

Helminth/tuberculosis (TB)-coinfection can reduce cell-mediated immunity against *Mycobacterium tuberculosis* (Mtb) and increase disease severity, although the effects are highly helminth species dependent. Mtb have long been ranked as the number one single infectious agent claiming the most lives. The only licensed vaccine for TB (BCG) offers highly variable protection against TB, and almost no protection against transmission of Mtb. In recent few years the identification of naturally occurring antibodies in humans that are protective during Mtb infection has reignited the interest in adaptive humoral immunity against TB and its possible implementation in novel TB vaccine design. The effects of helminth/TB coinfection on the humoral response against Mtb during active pulmonary TB are however still unclear, and specifically the effect by globally prevalent helminth species such as *Ascaris lumbricoides*, *Strongyloides stercoralis*, *Ancylostoma duodenale*, *Trichuris trichiura*. Plasma samples from smear positive TB patients were used to measure both total and Mtb-specific antibody responses in a Peruvian endemic setting where these helminths are dominating. Mtb-specific antibodies were detected by a novel approach coating ELISA-plates with a Mtb cell-membrane fraction (CDC1551) that contains a broad range of Mtb surface proteins. Compared to controls without helminths or TB, helminth/TB coinfected patients had high levels of Mtb-specific IgG (including an IgG1 and IgG2 subclass response) and IgM, which were similarly increased in TB patients without helminth infection. These data, indicate that helminth/TB coinfected have a sustained humoral response against Mtb at the level of active TB only. More studies on the species-specific impact of helminths on the adaptive humoral response against Mtb using a larger sample size, and in relation to TB disease severity, are needed.

**Supplementary Information:**

The online version contains supplementary material available at 10.1186/s13223-023-00808-0.

## Introduction

Tuberculosis (TB) caused by *Mycobacterium tuberculosis* (Mtb), account for approximately 10 million new cases yearly of TB and kills 1.5 million [1]. It is known that helminths and Mtb exhibit a high degree of co-existence mostly in endemic or underdeveloped countries. Approximately 1 billion people are infected with *Ascaris lumbricoides* and 240 million people are infected with *Schistosoma mansoni*. Helminths and Mtb are known to co-exist geographically; however both exert distinct but often opposing immune responses. Increasing evidence support that helminth infections can modulate the cell-mediated immune response to TB [2–6]. However, it is less clear how the humoral immune response against TB is affected during helminth coinfection, and so far only studied in murine models [7, 8] or during latent TB where Mtb-specific IgG and IgM levels were found decreased with helminth infection and rescued by anti-helminthic treatment [9].

While cell mediated immunity is well known for its contribution in protection against Mtb, adaptive humoral immunity is getting greater recognition for its protection [10–12]. Antibodies orchestrate protection through sophisticated mechanisms like neutralization of secreted antigen, opsonization and enhanced phagocytosis of bacteria, antibody-dependent cellular cytotoxicity (ADCC), and complement-dependent cytotoxicity. It is becoming clear that antibodies play a protective and beneficial role during latent TB infection [13], and serological evidence indicates that IgG, IgM and IgA are also detected during active TB [14, 15]. IgG is the most abundant antibody isotype in plasma and enables protection via complement activation and by enhancing macrophage phagocytosis of Mtb [16]. In line with IgG being important during infection, its subclasses IgG1, IgG2 and IgG3 was found to be involved in Mtb protection [17], and IgG1 and IgG2 predominant during active TB [18]. Antibodies from highly Mtb-exposed healthcare workers that provided protection against Mtb in a mouse infection model, had high levels of IgG1 and IgG3, and also showing presence of IgG2, IgA, and IgM [19]. In a whole blood assay, the protective effect of these antibodies was dependent on CD16/CD32A binding, CD4 T cells, and MHC class II, and were directed against the surface of Mtb.

Similar to IgG, IgA has shown to exhibit a protective role during Mtb infection. It was shown that IgA is involved in early protection against Mtb in humans [20] and for reducing Mtb susceptibility in mouse infection models as proven through IgA depletion or passive immunization with Mtb-specific IgA [21, 22]. However, increased levels of IgA correlate with the extent of disease in patients with pulmonary TB [23], indicating that IgA is also a sign of mucosal inflammation and enhanced disease severity. IgM has similar functions to IgG in that it opsonizes Mtb and can induce ADCC [24], additionally similar to the neutralizing effect of IgA it has also been shown to block Mtb attachment to epithelial cells by binding to heparin-binding hemagglutinin of Mtb [25]. Unlike IgG and IgA, IgM is present at high levels in the early stages of Mtb infection and diminishes during the advanced stage of TB. However, it is still largely unknown how infection with different helminth species modulates the protective humoral response against Mtb during coinfection.

In this study, we investigated how helminth infection modulates the total and Mtb-specific antibody levels during pulmonary TB. With our approach to detect Mtb-specific antibodies against the surface of Mtb in smear positive TB patients, we detect elevated levels of Mtb-specific antibodies with no differences between helminth negative TB patients and TB/helminth coinfected patients.

## Materials and methods

### Ethics statement

Written informed consent was obtained from all study participants. Ethical approval was obtained from the Institutional Committee on Research Ethics (CIEI) of the Peruvian University Cayetano Heredia.

### Study participants

TB patients and healthy individuals with or without helminth infection were recruited from Iquitos and Lima, Peru, and healthy individuals from Sweden served as non-endemic controls. All participants were between 18 and 79 years of age. TB and/or helminth infection status was used to categorize the participants into the three endemic groups, helminth negative TB-negative healthy (endemic control), helminth negative TB-positive (Helminth-/TB+), helminth positive TB-positive (Helminth+/TB+); and including non-endemic controls from Sweden (Sweden non-endemic control). For the purpose of this study, being the first of its kind analyzing Mtb-specific antibodies in patients with active pulmonary TB and helminth coinfection, helminth positive patients was included regardless of having single or multiple helminth infections (Supporting information S1 Fig), for all analyses presented herein. All helminth negative endemic controls and 23 TB patients without helminths were from Lima, whereas the remaining TB patients with or without helminths were from Iquitos, a soil-transmitted helminth (STH)-endemic area of the Peruvian Amazon. Newly diagnosed TB patients with acid fast bacilli (AFB) smear positivity and clinical signs of TB were considered for the active pulmonary TB-positive groups and included in the study if their sputum sample was also positive by the Microscopic Observation Drug Susceptibility (MODS) liquid culture test. For parasitological examination three stool samples from each participant were screened for helminth infection and helminth species identification by direct microscopy as well as microscopy after formol-ether concentration using Ritchie’s method and Ziehl-Neelsen staining. Inclusion criteria for TB-negative subjects included: no history of TB disease and showing none of the classical TB symptoms such as chronic cough with blood-tinged sputum, fever, or night sweats. Major exclusion criteria used for all four Peruvian subgroups: subjects that have been on anti-TB drugs for more than one week, recently on anti-helminth drug therapy, self-reported HIV positive, any underlying or current medical condition apart from TB or helminth infection (such as sepsis, diabetes mellitus, cancer etc.), and subject is pregnant.

### Quantification of total IgG, IgA, IgM, and CRP

Whole blood with sodium heparin as anticoagulant was the source of plasma for samples from Peru, and EDTA-plasma was used from Swedish controls. Total IgG, IgA, IgM, and C-reactive protein (CRP) in plasma was analysed by Clinical Chemistry, Laboratory Medicine at Linköping University Hospital, according to their routine analysis.

### Quantification of total IgE

Total IgE antibodies in plasma was analyzed using the Human IgE ELISA Kit according to manufacturers’ instructions (BMS2097, ThermoFisher Scientific, United Kingdom).

### Immunosorption to remove IgG using Eurosorb

Before quantification of Mtb-specific IgM and IgA, IgG was removed from plasma by immunoabsorption using eurosorb. The motivation being that high levels of IgG will bind specifically to the coated antigen and elicit false negative or low IgA and IgM results. IgG was removed according to manufactures instruction by diluting the plasma 1:10 with EUROSORB IgG/RF absorbent (EUROIMMUN AG), vortexing for 4 s, and incubation at room temperature for 15 min. After centrifugation at 2000 rpm for 5 min, these IgG-depleted plasma supernatants were used for determining Mtb-specific IgA and IgM. For Mtb-specific IgG, the untreated plasma was used.

### Detection of Mtb-specific antibodies

Mtb-specific antibodies in plasma was analysed using indirect ELISA, as previously described for purified protein derivative (PPD) of Mtb as antigen and other Mtb-antigens [26]. Microlon high binding microplates (Greiner Bio-One) were coated with 5 µg/ml PPD (Statens Serum Institute, Copenhagen, Denmark) or 5 µg/ml of cell membrane fraction from mycobacterium strain CDC1551 (NR-14,832 Mycobacterium tuberculosis Cell membrane fraction ATCC 10,801 University Blvd, Manassas, VA 20,110 − 2209, USA), in PBS for 2 h at 37 °C. Plates were washed thrice with phosphate-buffer saline (PBS) containing 0,05% Tween 20 (PBST), and blocked with PBS containing 1% Tween 20 and 1% bovine serum albumin (BSA) for 1 h at 37 °C. Plates were washed thrice and serially diluted plasma samples in PBST with 1% BSA (1:25, 1:200, 1:400, 1:800) were added and incubated over night at 4 °C. Plates were washed and incubated for 2 h at 37 °C with polyclonal rabbit anti-human IgG HRP (1:5000), polyclonal rabbit anti-human IgA HRP (1:4000), or polyclonal rabbit anti-human IgM HRP (1:1000), all obtained from Dako, Denmark A/S. Plates were washed and tetramethyl benzidine (TMB) substrate was added. Reactions were stopped with 1 M sulfuric acid and absorbance was measured at 450 nm. For IgG subclass determination of Mtb-specific antibodies, mouse monoclonal anti-human IgG1 HRP (ab99774, Abcam, United Kingdom), mouse monoclonal anti-human IgG2 HRP (ab99779, Abcam, United Kingdom), was used similarly as described above. The dilution of these antibodies was 1:1500 (IgG1), 1:1500 (IgG2).

For determining which antigens to use for screening of Mtb-specific antibodies we initially compared a purified protein derivative of Mtb (PPD) with a cell membrane fraction from Mtb (Mtb strain CDC1551), in the same ELISA using a limited number of plasma samples from patients and controls. In helminth negative PTB patients, there were higher levels of IgG1 and IgG2 against the Mtb cell membrane fraction than against PPD (Supporting information S2 Fig). This was not on account of higher levels of unspecific binding, as the background level from plasma of TB negative Peruvian controls was not noticeably affected when probing against the cell membrane fraction of Mtb compared to when probing plasma against PPD. SDS page result of the two proteins was consistent with this, showing that the cell membrane fraction of Mtb contained a broader range of Mtb-derived proteins whereas PPD was composed of a very limited number of proteins. Based on this pilot screening, and the fact that antibodies raised against the surface of Mtb are what have been found protective [19], we proceeded with the cell membrane fraction of Mtb (strain CDC1551) for screening of Mtb-specific antibodies.

### Statistics

Statistical analysis was performed using GraphPad Prism version 8. 4. 3. Based on results from normality test (shapiro wilk and kolmogorov smirnov test) total IgG, IgA, IgM and CRP significance testing was performed with a parametric test (One-way ANOVA) and Mtb-specific antibody levels significance testing was performed with a non-parametric test (Kruskal-Wallis test with Dunn’s post testing). The main comparison was against the endemic Peru control (shown by * in graphs). As a secondary analysis, all groups were compared against the non-endemic Sweden control (shown by # in graphs). p < 0.05 was considered significant.

## Results

### Characteristics of study population

47% of the helminth positive TB patients were infected by multiple helminth species (supporting information S1 Fig). Men dominated in both TB groups and endemic controls, while there were more women among non-endemic controls (Table 1). Non-endemic Swedish controls had a significant increase in the mean age compared to endemic control (p < 0.0245), whereas there were no differences in the mean age between the groups from Peru. CRP-levels were high in both TB-groups and significantly increased compared to endemic control, whereas remaining TB-negative groups showed negative-to-low levels of CRP indicating that they had no ongoing infection. Total IgE was significantly elevated in all endemic Peruvian groups compared to non-endemic Swedish controls as previously shown for Peruvians and different ethnic groups [27–29].

**Table 1:** Demographic of TB patients and controls

	Peru			Sweden
	Endemic	Helminth-	Helminth+	Non-endemic
	control	TB+	TB+	control
n	30	42	19	10
MODS	All neg.	All pos.	All pos.	NA
AFB	All neg.	All pos.	All pos.	NA
Helminth	All Helm. neg.	All Helm. neg.	All Helm. pos.	NA
Age Median (R)	27(18–76)	29.50(18–79)	33(19–70)	53.5(26–57)
p-value*		NS	NS	P = 0.0245
Gender Male (%)	18(60)	27(64.29)	13(68.42)	3(30)
p-value*		NS	NS	NS
CRP (mg/ml) Median (R)	1.475 (0.090–27.83)	53.65 (0.770–216.9)	52.09 (0.40–120)	0.530 (0.130–3.60)
p-value*		P = 0.0001	P = 0.0001	NS
Total IgE (ng/ml) Median (R)	236 (16.5–715)	273 (26.8–702)	367 (22.1–978)	25.3 (4.71–217)
p-value*		NS	NS	P = 0.0012

TB, tuberculosis; AFB, Acid Fast Bacilli; MODS, Microscopic Observation Drug susceptibility; R, Range; NA, not analyzed; neg, negative; NS, not significant; CRP, C reactive protein; P: significant difference to Peru control; n: numbers; Helm. neg, helminth negative; Helm. pos, helminth positive. *Statistics performed with Kruskal-Wallis test

### Increase in total IgG, IgA and IgM in plasma of helminth positive TB patients

**Fig. 1:**
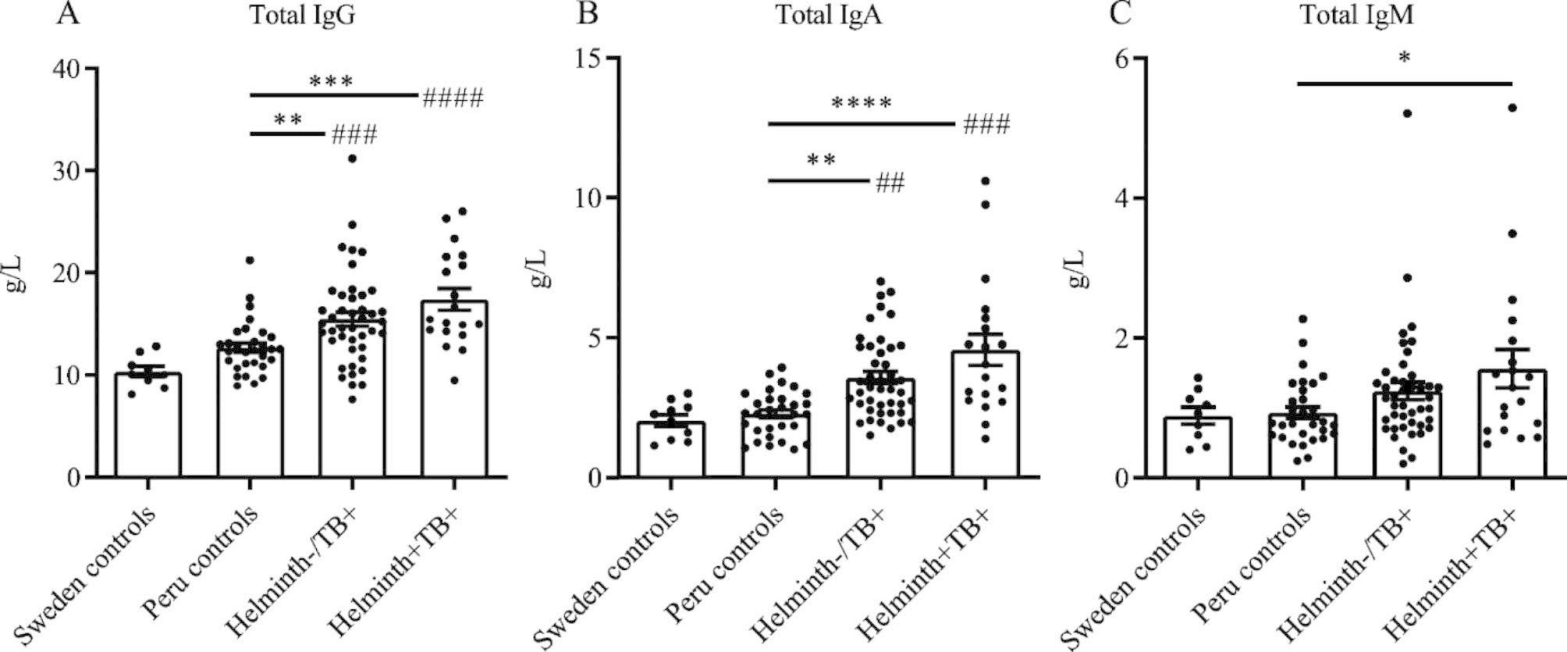
Total plasma IgG, IgA and IgM: Total plasma IgG, IgA, and IgM was measured in non-endemic Sweden control samples (n = 10), in Peruvian controls that were helminth negative and healthy (n = 30), in helminth negative pulmonary TB patients (Helminth-/TB+; n = 42), and in helminth positive pulmonary TB patients (Helminth+/TB+; n = 19). Data are presented as scatter plot bar graphs with each circle representing a single individual and bars depicting mean ± SEM. *, p < 0.05; **^/##^, p < 0.01; ***^/###^, p < 0.001; ****^/####^, p < 0.0001 using One-way ANOVA and asterisk sign (*) indicate significance to Peru control and number sign (#) indicate significance to Sweden control

Compared to healthy endemic control, TB patients without helminth infection had a significant increase in total IgG and IgA (Fig. 1). This IgG and IgA response was further elevated in helminth positive TB patients, who also exhibited a prominent and significantly elevated total IgM.

### Mtb cell membrane antigen-specific IgG, IgA, and IgM

**Fig. 2:**
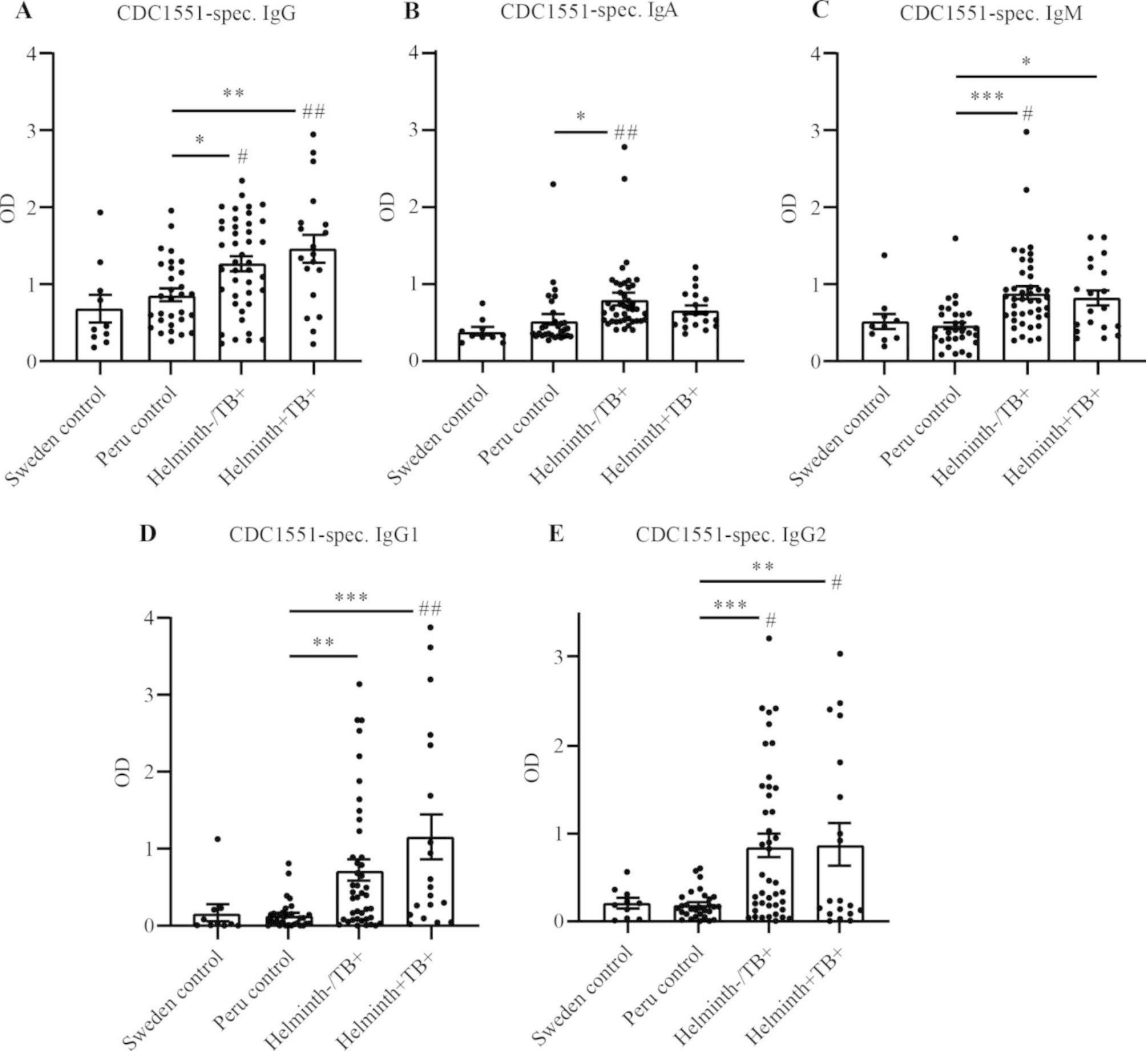
Mtb cell membrane-specific antibody response during helminth coinfection: Mtb-specific antibodies in plasma was analyzed using ELISA-plates coated with Mtb strain CDC1551 cell membrane fraction and HRP-conjugated anti-IgG (1/400 dilution of plasma) (**A**), anti-IgA (1/200 dilution of plasma) (**B**), anti-IgM (1/200 dilution of plasma) (**C**), anti-IgG1 (1/400 dilution of plasma) (**D**), anti-IgG2 (1/200 dilution of plasma) (**E**). Plasma was from non-endemic Sweden control samples (n = 10), Peruvian controls that were helminth negative and healthy (n = 30), helminth negative pulmonary TB patients (Helminth-/TB+; n = 42), and helminth positive pulmonary TB patients (Helminth+/TB+; n = 19). Data are presented as scatter plot bar graphs with each circle representing a single individual and bars depicting mean ± SEM of the OD_450_-value after background subtraction of Mtb strain CDC1551 cell membrane fraction-coated wells receiving wash buffer instead of plasma but otherwise treated the same. *^/#^, p < 0.05; **^/##^, p < 0.01; ***, p < 0.001 using One-way ANOVA and asterisk sign (*) indicate significance to Peru control and number sign (#) indicate significance to Sweden control

Next, we evaluated the presence of Mtb-specific antibodies using a cell membrane fraction of Mtb (Mtb strain CDC1551), which specifically detects antibodies against the surface of Mtb [30]. We observed a significant increase in Mtb cell membrane specific IgG, and IgM in helminth/TB coinfected patients compared to endemic controls however only IgG was significantly increased compared to non-endemic controls (Fig. 2). TB patients without helminths had a significant increase in Mtb cell membrane specific IgG, IgM, and IgA compared to endemic and non-endemic controls. Further analysis of the IgG subclass response against the cell membrane antigens of Mtb showed a significant increase in IgG1 and IgG2 for helminth/TB coinfected patients, both when compared to endemic and non-endemic controls. For TB patients without helminth infection, IgG1 and IgG2 were also significantly increased. Our pilot screening showed low and non-significant levels of Mtb-specific IgG3 and IgG4 (Supporting information S3 Fig), in agreement with IgG1 and IgG2 being the predominant isotypes during active TB [18]. There was no significant difference in the levels of Mtb-specific antibodies between TB patients without helminth infection and helminth/TB coinfected patients, despite our approach using this cell membrane fraction of Mtb which has a broad range of Mtb-derived surface proteins (Supporting information S2 Fig).

## Discussion

There is increasing evidence of a beneficial role of adaptive humoral immunity in host immunity against TB [10, 12, 31]. In this study we investigated the effect of helminth on the Mtb-specific humoral response against TB. Our results show that the levels of Mtb-specific IgG, was significantly increased in smear positive TB patients compared to the endemic control, and this level of Mtb-specific IgG was not altered in helminth/TB coinfected individuals. This response was comprised of a strong IgG1 and IgG2 subclass induction. Helminth coinfected TB patients further had a substantial increase in both total IgM and Mtb-specific IgM. In contrast, Mtb-specific IgA levels were only increased in TB patients without helminth. Thus our data are consistent with previous studies showing that active TB is associated with an increased antibody response [10, 12, 32] and further indicate that TB patients with helminth coinfection show a clear antibody response against Mtb.

It is known that IgG against Mtb-specific proteins are abundant in individuals with latent TB as well as in patients with active TB [33]. Furthermore, increased levels of IgG have been correlated to increased protection against Mtb [34, 35]. Mtb-specific IgG also correlates with the absence of miliary TB in children emphasizing the protective role of Mtb-specific IgG [36]. Consistently our data show an increase in both total and Mtb-specific IgG in TB patients, with no difference with and without helminth/TB coinfection. Together these findings of increased or maintained IgG levels during helminth/TB coinfection indicate that helminths might not alter Mtb-specific antibodies and may have a protective role in the host during Mtb infection. However, *Strongyloides stercoralis* infection in individuals with latent TB showed reduced levels of Mtb-specific IgG and IgM that increased after anti-helminthic treatment [9] indicating that different helminth species can have quite opposing effects on the humoral immunity against Mtb.

Our study is the first to report on Mtb-specific IgA responses in helminth TB coinfection. We show that helminth/TB coinfected patients although having a high total IgA level showed low and non-significant levels of Mtb-specific IgA, in relation to TB patients without helminths that instead had significantly increased levels of Mtb-specific IgA compared to healthy controls. Previous studies have shown that IgA is involved in early pulmonary protection against mycobacterial infection in mice [21, 22, 37]. Human studies report that the presence of IgA during Mtb infection indicates a recent infection while others state that elevated IgA levels is an effect of extensive, active and miliary TB [37–39]. As all our patients were smear positive and had active pulmonary TB and therefore not likely recently infected by Mtb, the level of Mtb-specific IgA in our case would rather correlate to the extent or severity of infection. For other diseases it was reported that increased serum IgA is a biomarker of increased chronic hepatitis B infection [40], and for COVID-19 disease SARS CoV-19 specific IgA correlate with severity and critical illness [41]. Decreased levels of Mtb-specific IgA in the helminth/TB coinfected individuals could therefore be linked to a less severe or less disseminated TB with reduced mucosal inflammation, although further immunological findings and correlations to disease severity are needed to substantiate this. It has been reported that Mtb-specific IgA, to several antigens of Mtb, can distinguish between active TB and latent TB and Mtb non-infected healthy individuals, proposing serological identification of Mtb-specific IgA as a tool in diagnosing TB [42–45]. Our finding of a serological negative Mtb-specific IgA response in pulmonary confirmed TB patients with concurrent helminth infection, therefore, poses a general threat to such diagnostic efforts in helminth endemic regions and warrants further investigations on the effect of helminths on the adaptive humoral response against Mtb.

Increasing evidence shows that IgM can induce complement activation and engage in protection against Mtb [13] and that IgM levels are increased during latent TB infection. But in contrast to IgG and IgA, IgM is present at low levels during active TB [46] being produced mainly during the early phase of Mtb infection and declining in the advanced phase due to class switching to IgG [47]. The presence of IgM thus usually indicates an early and ongoing infection [15, 25, 38]. In line with this, our data also show a low level of total IgM in TB patients without helminths, but at the same time they have increased levels of Mtb-specific IgM. However, helminth/TB coinfected individuals had significantly increased levels of both total IgM and Mtb-specific IgM, despite them also exhibiting high levels of IgG. Our findings that IgM levels are increased during helminth/TB coinfection could be interpreted as that helminth coinfection is involved in the response limiting the transition to advanced TB, or a failure to establish class switching of IgM. Regardless, the constant expression and production of both Mtb-specific IgM and IgG during helminth coinfection may indicate enhanced or sustained protection against Mtb.

We analyzed Mtb-specific IgG subclasses to better understand the response during helminth/TB coinfection. IgG1, and IgG2 are the most prevalent IgG subclasses observed during active TB and observed against different Mtb antigens [18]. IgG1 is the most abundant, IgG2 is moderately expressed, whereas the IgG3 and IgG4 responses is negligible [48] as also indicated by our pilot screening of Mtb-specific IgG3 and IgG4 (supporting information S3 Fig). In agreement with previous studies, our data show that the levels of Mtb-specific IgG1 and IgG2 were increased in TB patients without helminths. Our data additionally suggest that, during helminth/TB coinfection, there is also a strong and pronounced IgG1 and IgG2 response raised against the surface of Mtb.

The gel electrophoresis data (supporting information S2 C Fig) along with a previous report [30] show that PPD (derived from the culture filtrate of Mtb) consists of a low range of Mtb-specific proteins, whereas the CDC1551 cell membrane fraction of Mtb is comprised of a broader range of Mtb-specific proteins. This could be the reason for the increased detection of Mtb-specific IgG1 and IgG2 when probing a limited set of clinical samples against the cell membrane fraction of Mtb, compared to when using PPD (as illustrated in supporting information S2 A-B Fig). More importantly, the IgG1 and IgG2 signals against the cell membrane fraction of Mtb, when using the complete set of clinical plasma samples were significantly elevated in helminth/TB coinfected patients compared to levels in endemic control, with no observed differences between helminth negative and helminth positive TB patients, despite our approach using this cell membrane fraction with a broad range of Mtb surface proteins.

Our cohort of TB patients were highly contagious smear positive TB patients verified by culture positivity, and helminth positive patients had asymptomatic helminth infection, which is associated with having a low worm burden [49]. Limitations of the study, was the sample size and the circumstance that TB patients at this helminth endemic site of the Peruvian Amazon were commonly infected with several helminth species (multiple helminth infection) making it difficult to interpret which helminth drives the response. It will be essential to verify these findings in larger cohorts of helminth infected individuals by analyzing the effect of single helminth infections and if possible, correlate such findings to the disease severity of TB, to more clearly delineate how the level of protection against Mtb by the adaptive humoral immunity is influenced by helminth coinfection. Future studies should consider, correlating the antibody response to worm burden, chest X-ray findings and TBscore [50] to assess the disease severity, as well as the inclusion of follow-up measurements after deworming.

To our knowledge, this is the first study examining the impact of helminth infection on the Mtb-specific antibody response during active, pulmonary confirmed TB. The increased levels of Mtb-specific IgG (IgG1, IgG2) and IgM in helminth coinfected TB patients suggest a maintained protective humoral response against TB, whereas the reduction in Mtb-specific IgA indicates reduced inflammation or induction of an anti-inflammatory or regulatory T and B cell response.

## Electronic supplementary material

Below is the link to the electronic supplementary material.

Supplementary Material 1: **Supporting information S1 fig. Helminth species distribution in the helminth + TB + group:** Of the helminth/TB coinfected group, 53% were infected with a single helminth and 47% were infected with multiple helminths. 15(80%) out of 19 helminth coinfected TB patients had *Trichuris trichiura* (TT) followed up by *Ascaris lumbricoides* (AL) 5(30%), *Strongyloides stercoralis* (SS) 4(20%), *Ancylostoma duodenale* (AD) 6(30%), and *Hymenolepis nana* (HN) 1(5%)

Supplementary Material 2: **Supporting information S2 Fig. Higher Mtb-specific IgG1 and IgG2 response against Mtb cell membrane fraction than against PPD in plasma of Peruvian TB patients**: Mtb-specific antibodies in plasma was analyzed using ELISA-plates coated with purified protein derivative from Mtb (PPD) or Mtb strain CDC1551 cell membrane fraction (CDC1551), and using either HRP-conjugated anti-IgG1 (1/800 dilution of plasma) (**A**), or HRP-conjugated anti-IgG2 (1/400 dilution of plasma) (**B**). Plasma was from Peruvian controls that were helminth negative and healthy (n = 15), and helminth negative pulmonary TB patients (Helminth-/TB+; n = 9). Data are presented as scatter plot bar graphs with each symbol (circle, PPD; triangle, CDC1551) representing a single individual and bars depicting mean ± SEM of the OD_450_-value after background subtraction of antigen-coated wells receiving wash buffer instead of plasma but otherwise treated the same. (**C**) Gel electrophoresis for separation of the Mtb antigens was performed on a 10% SDS-polyacrylamide gel and total protein evaluated by silver staining. 7.5 µg of each antigen was loaded onto the gel. Lane 1: CDC1551 cell membrane fraction of Mtb. Lane 2: purified protein derivative from Mtb (PPD). Lane 3: protein standard from Bio-Rad

Supplementary Material 3: **Supporting information S3 fig. Mtb cell membrane-specific IgG3 and IgG4 screening in peruvian TB patients:** Mtb-specific antibodies in plasma was analyzed using ELISA-plates coated with Mtb strain CDC1551 cell membrane fraction and HRP-conjugated anti-IgG3 (1/100 dilution of plasma) (**A**) and anti-IgG4 (1/25 dilution of plasma) (**B**). Plasma was from non-endemic Sweden control samples (n = 10), Peruvian controls that were helminth negative and healthy (n = 15), helminth negative pulmonary TB patients (Helminth-/TB+; n = 9), and helminth positive pulmonary TB patients (Helminth+/TB+; n = 8). Data are presented as scatter plot bar graphs with each circle representing a single individual and bars depicting mean ± SEM of the OD_450_-value after background subtraction of Mtb strain CDC1551 cell membrane fraction-coated wells receiving wash buffer instead of plasma but otherwise treated the same. Significance testing using One-way ANOVA showed no significance in TB groups compared to either Sweden control or Peru control

## Data Availability

All data generated or analysed during this study are included in this published article [and its supplementary information files].
