# Supplementary figures and images for: No impact of helminth coinfection in patients with smear positive tuberculosis on immunoglobulin levels using a novel method measuring Mycobacterium tuberculosis-specific antibodies
Source: Allergy Asthma Clin Immunol. 2023 Jun 29;19:55. doi: 10.1186/s13223-023-00808-0 (PMC10308675; doi:10.1186/s13223-023-00808-0)

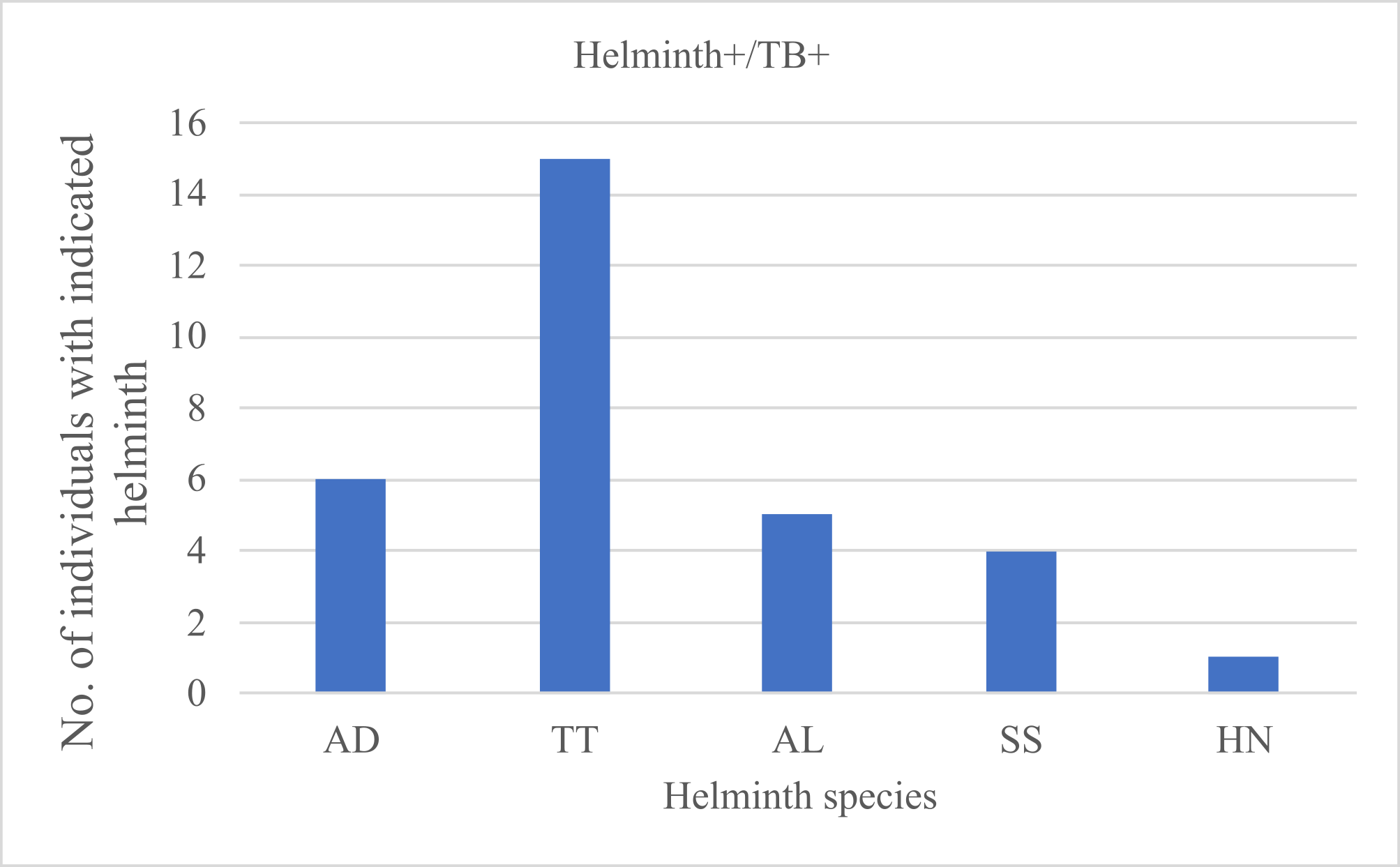

Supplement: Supplementary file 1 — Supplementary Material 1: Supporting information S1 fig. Helminth species distribution in the helminth + TB + group: Of the helminth/TB coinfected group, 53% were infected with a single helminth and 47% were infected with multiple helminths. 15(80%) out of 19 helminth coinfected TB patients had Trichuris trichiura (TT) followed up by Ascaris lumbricoides (AL) 5(30%), Strongyloides stercoralis (SS) 4(20%), Ancylostoma duodenale (AD) 6(30%), and Hymenolepis nana (HN) 1(5%) [file 13223_2023_808_MOESM1_ESM.png]

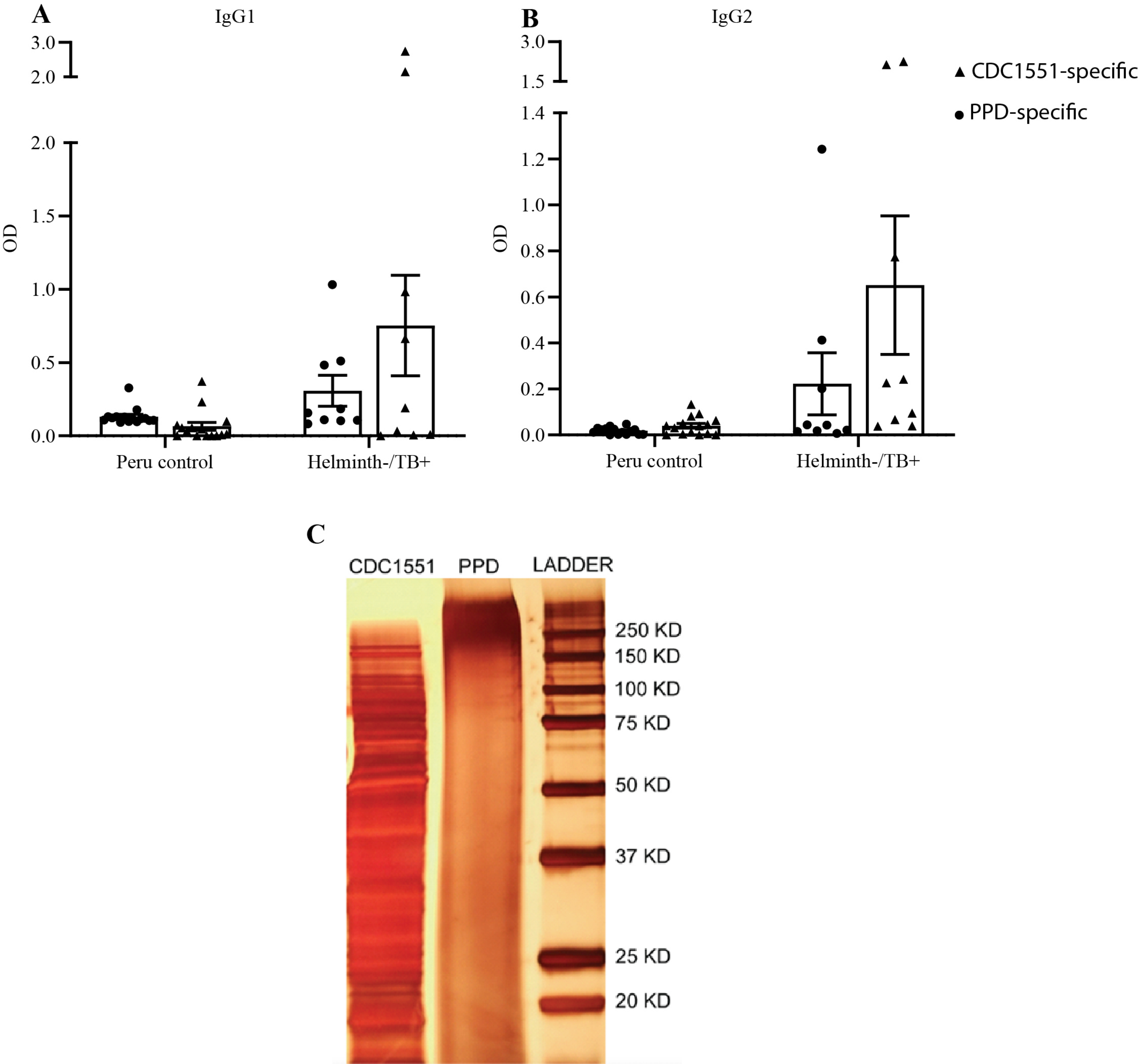

Supplement: Supplementary file 2 — Supplementary Material 2: Supporting information S2 Fig. Higher Mtb-specific IgG1 and IgG2 response against Mtb cell membrane fraction than against PPD in plasma of Peruvian TB patients: Mtb-specific antibodies in plasma was analyzed using ELISA-plates coated with purified protein derivative from Mtb (PPD) or Mtb strain CDC1551 cell membrane fraction (CDC1551), and using either HRP-conjugated anti-IgG1 (1/800 dilution of plasma) (A), or HRP-conjugated anti-IgG2 (1/400 dilution of plasma) (B). Plasma was from Peruvian controls that were helminth negative and healthy (n = 15), and helminth negative pulmonary TB patients (Helminth-/TB+; n = 9). Data are presented as scatter plot bar graphs with each symbol (circle, PPD; triangle, CDC1551) representing a single individual and bars depicting mean ± SEM of the OD450-value after background subtraction of antigen-coated wells receiving wash buffer instead of plasma but otherwise treated the same. (C) Gel electrophoresis for separation of the Mtb antigens was performed on a 10% SDS-polyacrylamide gel and total protein evaluated by silver staining. 7.5 µg of each antigen was loaded onto the gel. Lane 1: CDC1551 cell membrane fraction of Mtb. Lane 2: purified protein derivative from Mtb (PPD). Lane 3: protein standard from Bio-Rad [file 13223_2023_808_MOESM2_ESM.png]

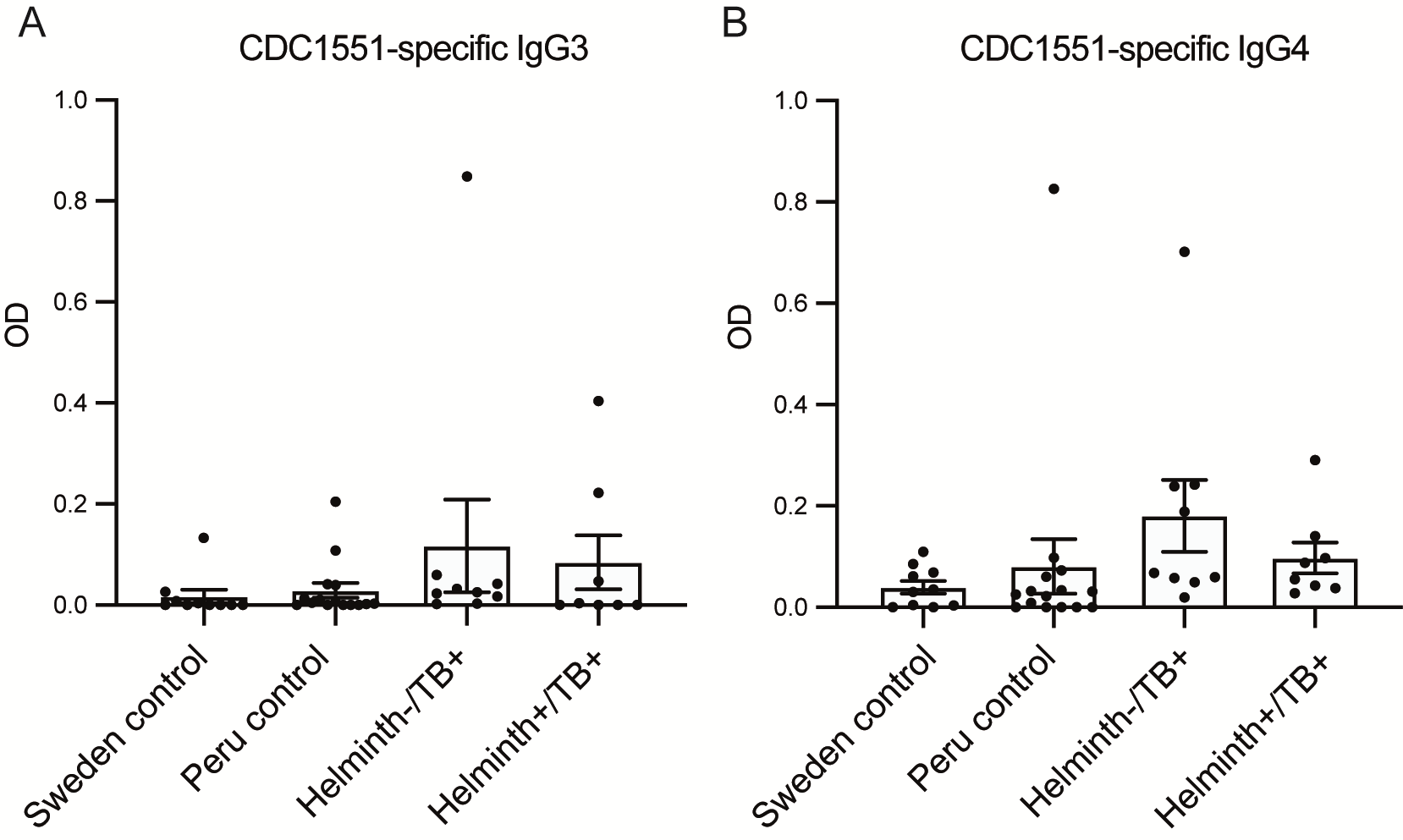

Supplement: Supplementary file 3 — Supplementary Material 3: Supporting information S3 fig. Mtb cell membrane-specific IgG3 and IgG4 screening in peruvian TB patients: Mtb-specific antibodies in plasma was analyzed using ELISA-plates coated with Mtb strain CDC1551 cell membrane fraction and HRP-conjugated anti-IgG3 (1/100 dilution of plasma) (A) and anti-IgG4 (1/25 dilution of plasma) (B). Plasma was from non-endemic Sweden control samples (n = 10), Peruvian controls that were helminth negative and healthy (n = 15), helminth negative pulmonary TB patients (Helminth-/TB+; n = 9), and helminth positive pulmonary TB patients (Helminth+/TB+; n = 8). Data are presented as scatter plot bar graphs with each circle representing a single individual and bars depicting mean ± SEM of the OD450-value after background subtraction of Mtb strain CDC1551 cell membrane fraction-coated wells receiving wash buffer instead of plasma but otherwise treated the same. Significance testing using One-way ANOVA showed no significance in TB groups compared to either Sweden control or Peru control [file 13223_2023_808_MOESM3_ESM.png]
